# Structural and lithological controls on landfill site suitability in Tenth of Ramadan City, Egypt using remote sensing and machine learning

**DOI:** 10.1038/s41598-026-41772-0

**Published:** 2026-03-23

**Authors:** Salma Essam, Walid M. Mabrouk, Khaled S. Soliman, Ahmed Metwally

**Affiliations:** Geophysics Department, Faculty of Science, Cairo University, Giza, 12613 Egypt

**Keywords:** Remote sensing, Principle Component Analysis (PCA), K-means unsupervised classification, Support Vector Machine (SVM) supervised classification, Structural lineaments, Engineering, Environmental sciences, Solid Earth sciences

## Abstract

The study area, located east of Tenth of Ramadan City, represents a vital urban center on the desert fringes of the eastern Nile Delta. In accordance with governmental regulations for landfill construction in new developments, this study aims to identify optimal landfill sites by integrating environmental and geotechnical considerations. Remote sensing and machine learning techniques (K-means and Support Vector Machine (SVM)) were applied to Landsat 5 TM imagery for lithological mapping. The SVM classification achieved an overall accuracy of 82.49% and a Kappa coefficient of 0.7235, providing a significant spatial refinement over the regional geological map by identifying localized lithological variations and recent urban expansion. Additionally, structural analysis using PCA yielded a 25% increase in detected lineament lengths 19.3 km compared to legacy data 15.4 km. Furthermore, ASTER GDEM data were used to generate a digital elevation model to visualize topographic variations and support structural analysis. The results revealed clear delineation of lithological units and highlighted zones of high lineament density, with dominant NE–SW (Syrian Arc) and NW–SE (Clysmic) structural trends influencing hydrogeological and geotechnical stability. Multi-criteria decision analysis (MCDA) was employed to map suitability, indicating that approximately 16.2% of the study area is highly suitable for landfill siting. These findings provide a practical framework for urban planners, providing a reliable decision-support framework for landfill selection based on integrated lithological and structural evidence.

## Introduction

Fractures and faults are key structural features that control groundwater movement and storage^1,2^. In addition to their importance in hydrogeology, structural mapping contributes to various disciplines, including site selection for infrastructure projects (dams, bridges, and roads), seismic and landslide hazard assessment, mineral exploration, tectonic geomorphology, and structural geological studies^3–5^. Therefore, accurate mapping of these features is essential for both environmental and engineering applications, especially in arid and semi-arid regions where surface indicators are often limited. Recent studies have emphasized the importance of integrating structural, geomorphic, and tectonic analyses through remote sensing and geophysical approaches to evaluate active deformations and overcome the limitations of traditional field mapping, particularly in data-scarce environments^6,7^.

Remote sensing techniques provide an efficient and cost-effective analysis and mapping of surface geology, providing a synoptic vision of areas that are often difficult to study from ground observations^8,9^. In recent years, numerous studies have demonstrated the value of integrating remote sensing data with geophysical techniques, field observations or geological maps to enhance lineament detection and structural interpretation^10,11^.

The present study focuses on an area east of Tenth of Ramadan City, Egypt, which is characterized by sedimentary formations affected by a set of structural features, posing significant geotechnical challenges for urban and industrial expansion. Consequently, precise mapping of these lithological and structural lineaments is critical for ensuring sustainable land-use planning and long-term infrastructure stability in this region.

While various remote sensing-GIS-MCDA frameworks have been applied to landfill site selection, a significant research gap remains in the high-precision integration of machine learning-derived lithology with automated structural density analysis in desert-fringe urban areas. This study addresses this gap by implementing a multi-stage workflow that refines traditional geological maps using SVM classification and ASTER-derived terrain gradients. The primary objective is to develop an integrated framework that (i) enhances lithological discrimination using machine learning, (ii) extracts and analyzes structural lineaments to reveal previously unmapped features, and (iii) integrates these refined geological factors into a GIS-based MCDA model. By providing a more rigorous geotechnical foundation, this approach enhances the reliability of the suitability index in semi-arid regions where topographic contrast is minimal, ultimately supporting environmentally sound landfill site selection in Tenth of Ramadan City.

## Study area and geological setting

**Fig. 1:**
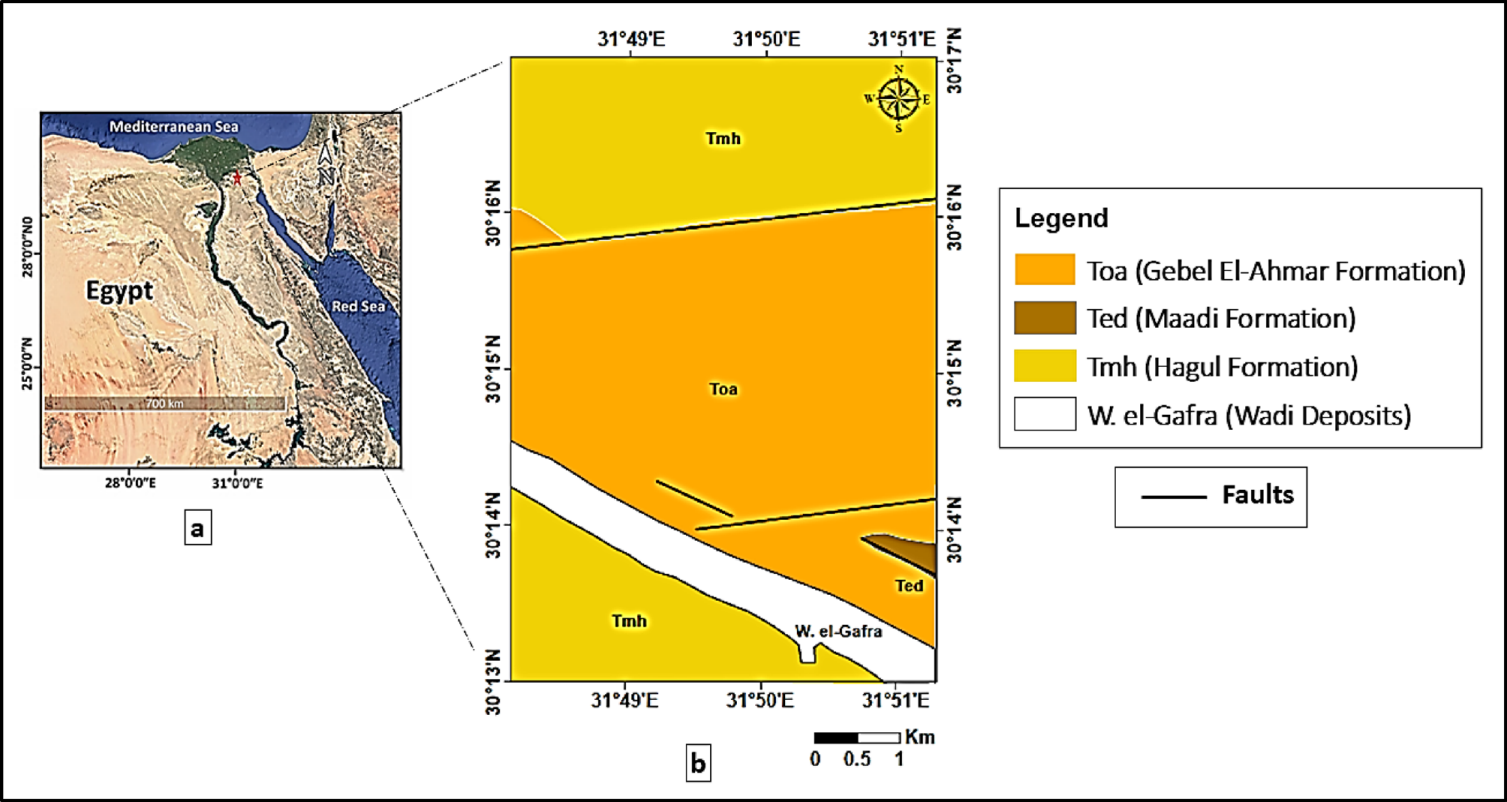
(**a**) Location map of the study area east of 10th of Ramadan City, Egypt. (**b**) Geological map of the study area illustrating the main lithostratigraphic units and major fault lines (modified after Conoco Coral, 1987; scale 1:500,000).

The concerned study area is located to the east of 10th of Ramadan City, Eastern Desert of Egypt (Fig. 1a), approximately between 30° 13′ 22′′ and 30° 17′ 27′′ N, and 31°48′ and 31° 51′ 55′′ E covering a total area of approximately 31 square kilometers. The climatic setting of the 10th of Ramadan area is characterized by a semi-arid to arid desert climate, featuring hot, dry summers and mild winters. The mean annual precipitation is approximately 20 mm, with a peak of 5 mm in January, while the driest months experience negligible rainfall. The temperature averages fluctuate between 12.9 °C in January and 28.3 °C in August, though summer maximums can reach significantly higher values. Relative humidity ranges from 39.5% in May to 55.2% in December, reflecting the dry nature of the air. Evaporation rates are notably high, ranging from (4.3 mm/day at Belbies metrological station) to (14 mm/day at Cairo metrological station), which exceeds the precipitation intensity^12^ and significantly minimizes the potential for continuous leachate generation.

Geologically, the area has prominent surface structural lineaments, which are suitable for detection and interpretation using remote sensing techniques. The geological map of the study area (Fig. 1b) shows four principal lithostratigraphic units ranging in age from the Upper Eocene to Recent. The Upper Miocene Hagul Formation (Tmh) dominates the northern and southwestern parts of the study area and consists primarily of fluvial sand and gravel, underlain by Hammath Fm. which consists of marine fossiliferous limestone with sandy layers at the base, locally underlain by white limestone with marl (Sadat Fm.)^13^. The stratigraphic succession reflects a clear a transition from marine to continental fluvial environments.

The Oligocene Gebel El-Ahmar Formation (Toa) occupies the central to southern parts of the study area, and consists of vividly colored continental sands, quartzite, and gravel, indicating deposition in a high-energy fluvial environment. The Upper Eocene Maadi Formation (Ted), observed at the southeastern edge of the area, consists of littoral and shallow shale and limestone with *carolia placunoides* locally intercalated with shallow marine sandstone coeval with Qasr el-Sagha Fm.)^13^, which suggests deposition in a shallow marine shelf environment.

Cutting across these stratigraphic units is Wadi el-Gafra, represented by recent unconsolidated wadi deposits composed of sand, silt, and scattered gravels, filling topographic lows and aligning along tectonic structures. The lithological descriptions and stratigraphic nomenclature of these formations were adopted from the published geological map of Egypt^14^, issued by the Egyptian General Petroleum Corporation (EGPC).

The structural framework of the study area was initially identified from the published geological map^15^, which highlights several fault lines affecting the Miocene, Oligocene, and Eocene formations. To refine this framework, both classical and recent interpretations were considered, revealing dominant structural trends in E–W and NW–SE directions, consistent with the mobile belt zone^15–17^.

### Data and methodology

**Fig. 2:**
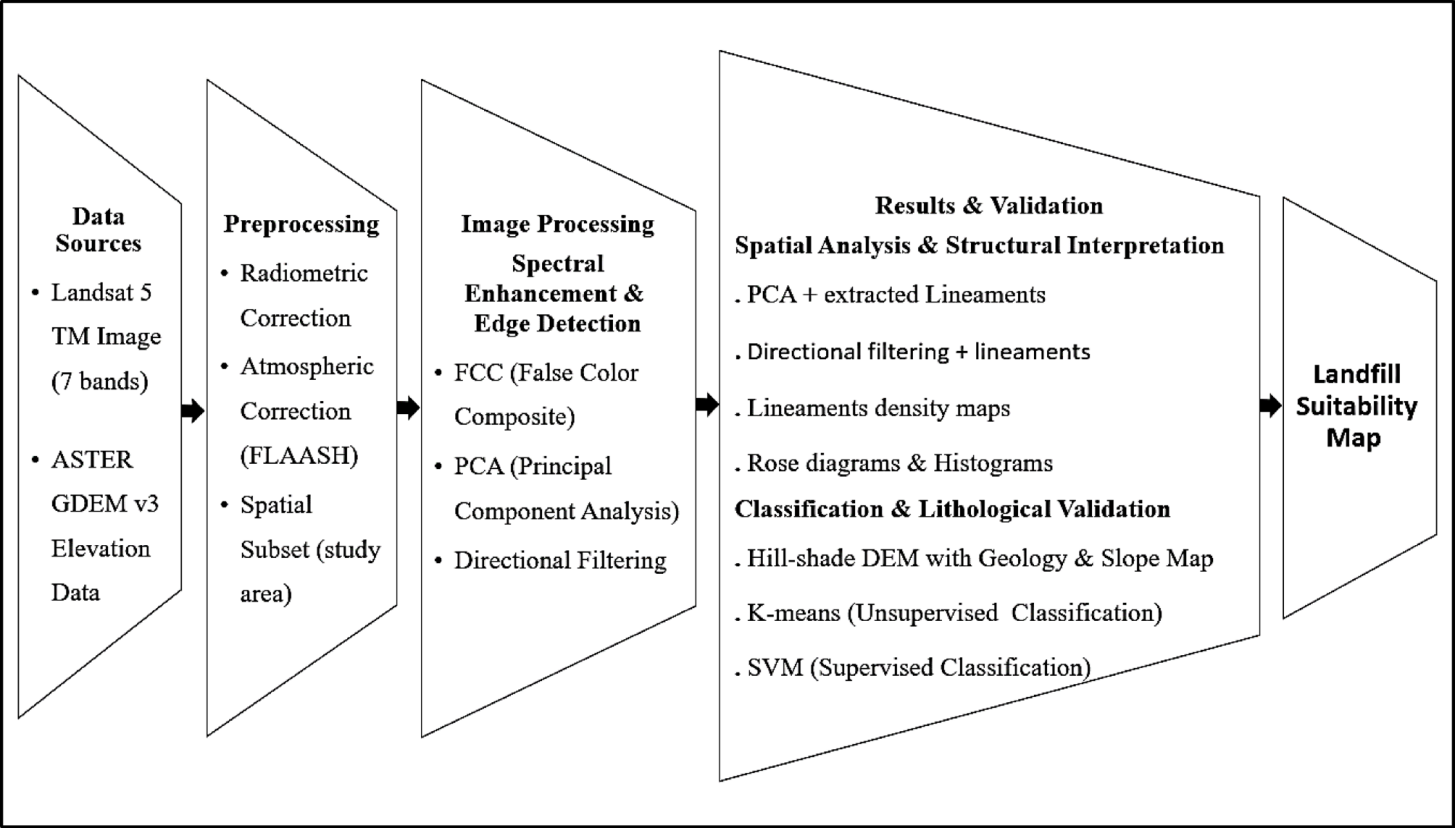
Workflow diagram illustrating the main steps followed in the remote sensing–based lithological and structural analysis.

The methodology adopted in this study integrates satellite imagery processing, DEM analysis, and structural feature extraction for lithological and structural investigation. Various specialized software packages were employed to perform these tasks, as detailed in (Table 1). The overall workflow is summarized in (Fig. 2).

**Table 1:** Software used and their applications in this study.

Software	Application
ENVI 5.3+	Radiometric & Atmospheric corrections FCC, PCA, and Directional filtering k-means, and SVM classifications
Global Mapper	Clipping study area and exporting elevation data (ASTER GDEM v3)
Surfer	Generation Hill-shaded color relief map from DEM
PCI Geomatica Banff	Lineaments extraction from PCA, and directional filtered images
ArcMap 10.7.1	Final map integration, layout design, spatial analysis, and preparation of the modified geological map
ArcGIS Pro	Lineament density mapping, Histograms and Weighted Overlay analysis for generating the landfill suitability map
QGIS Desktop 3.34.1	Rose diagrams

Portions of the text were refined with the assistance of ChatGPT (OpenAI, San Francisco, CA, USA) to improve clarity and language, with all content subsequently reviewed and verified by the authors.

## Data sources

### Landsat 5 TM imagery

A multispectral satellite image acquired by the Landsat 5 Thematic Mapper (TM) sensor on August 5, 2002 was used in this study. The image corresponds to WRS-2 Path 176, Row 039. The dataset is characterized by zero cloud cover, with an overall image quality score of 7. The spectral and spatial characteristics of the Landsat 5 TM bands utilized for this analysis are summarized in (Table 2). The spatial resolution is 30 m for both reflective and thermal bands. The data is projected using the Universal Transverse Mercator (UTM) system, zone 36 North, based on the WGS84 datum and ellipsoid.

This scene was selected due to its high spatial and radiometric quality, minimal atmospheric interference, and optimal coverage of the structural and lithological features within the study area. Although Landsat 7 (ETM+) was also operational at that time, the Landsat 5 TM data were preferred because they provided a complete, gap-free image with stable radiometric calibration, ensuring consistent results for subsequent analysis.

**Table 2:** Spectral and spatial characteristics of Landsat 5 TM bands used in this study.

Band	Spectral range (µm)	Description	Spatial resolution (meters)
1	0.45–0.52	Blue	30
2	0.52–0.60	Green	30
3	0.63–0.69	Red	30
4	0.76–0.90	Near Infrared (NIR)	30
5	1.55–1.75	Shortwave Infrared (SWIR 1)	30
6	10.40–12.50	Thermal Infrared (TIR)	120 (resampled to 30)
7	2.08–2.35	Shortwave Infrared (SWIR 2)	30

### ASTER GDEM v3 elevation data

ASTER GDEM v3 (30 m resolution) was utilized to generate a Digital Elevation Model (DEM) for the study area. The area of interest was extracted using Global Mapper and exported as an XYZ grid file. Then this file was imported into Surfer software to create a color relief map. Hill-shade enhancement was applied by adjusting azimuth and altitude parameters according to the solar angles of 10th of Ramadan City. This process significantly improved the visualization of structural and geomorphological elements, aiding in the preliminary identification of lineaments and structural features, such as potential faults and wadi systems.

### Image preprocessing

Prior to analysis, the acquired Landsat 5 TM imagery underwent a series of preprocessing steps to enhance data quality and ensure the accuracy of subsequent image interpretation. The first step of radiometric correction involved converting the pixel values (Digital Numbers) into at-satellite radiances, followed by correction for atmospheric propagation effects to obtain at surface radiances^18^.

Atmospheric correction was conducted using the FLAASH (Fast Line-of-sight Atmospheric Analysis of Spectral Hypercubes) module implemented in the ENVI 5.3 software. This correction approach is based on the MODTRAN (MODerate Resolution Atmospheric Transmission) radiative transfer model, which simulates the interaction of solar radiation with atmospheric constituents such as gases, aerosols, and water vapor^19^. MODTRAN enables accurate estimation and removal of atmospheric effects, including scattering and absorption, by modeling how light travels through the atmosphere under specified conditions. The FLAASH correction significantly improved the spectral integrity of the image by converting the sensor radiance to surface reflectance^19^.

Additionally, a spatial subset corresponding to the study area was extracted using ENVI to reduce processing time and focus on relevant geological features.

### Image processing and structural feature extraction

The Landsat 5 Thematic Mapper (TM) dataset consists of seven spectral bands covering the visible, near-infrared, shortwave infrared, and thermal infrared regions. In this study, only the six reflective bands (Bands 1–5 and 7) were selected for spectral enhancement and geological interpretation. The thermal band (Band 6), which has a coarser spatial resolution (120 m resampled to 30 m), was excluded due to its limited applicability in lithological discrimination and structural analysis^20^. To enhance the ability to distinguish lithological units and structural lineaments, the selected six bands were used to produce a False Color Composite (FCC) and perform Principal Component Analysis (PCA). The image processing tasks were carried out using ENVI 5.3 software, while post processing, and map production were completed using ArcGIS 10.7.1.

**Fig. 3:**
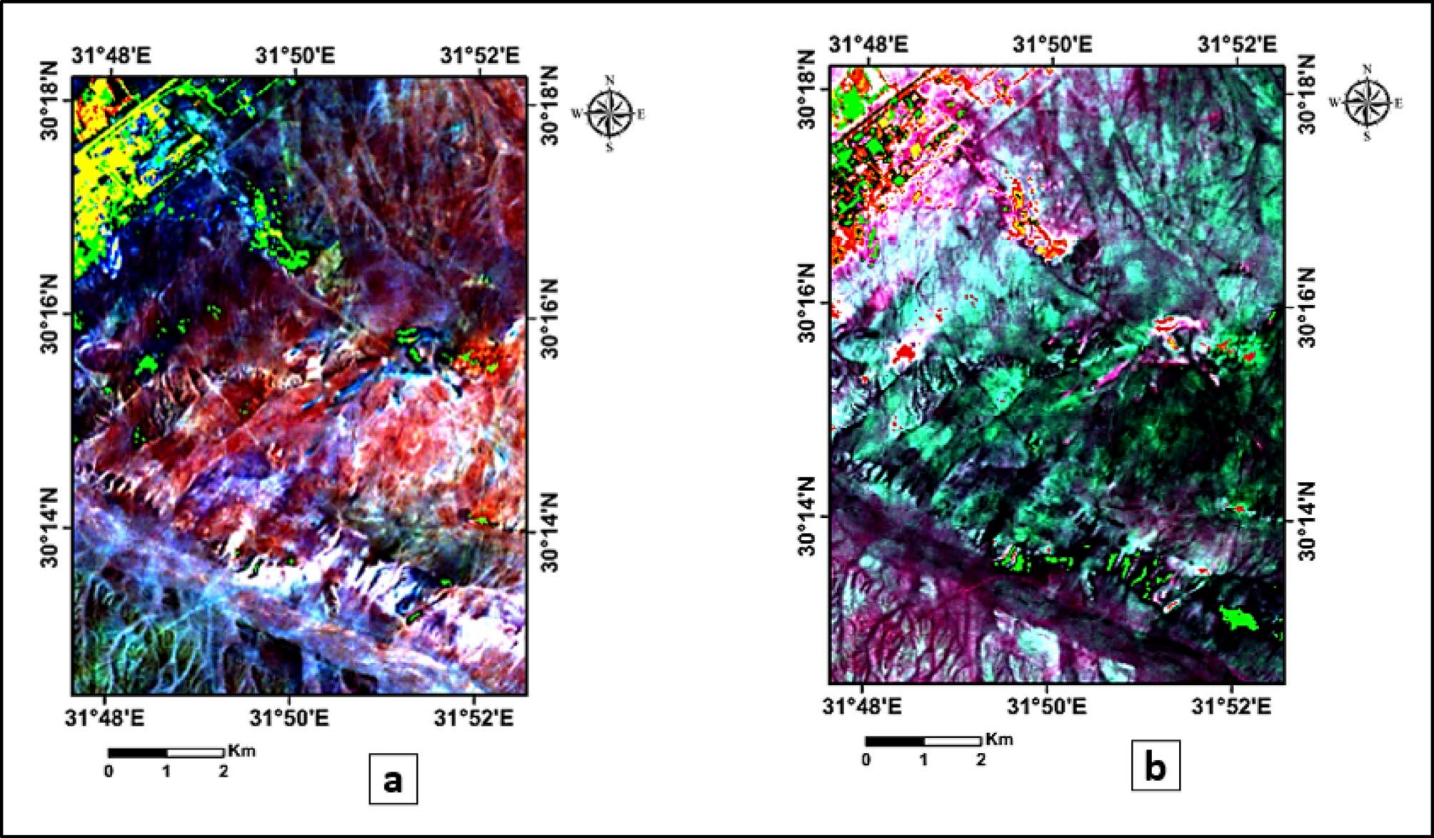
(**a**) False Color Composite (FCC) of Landsat 5 TM (Bands 7, 4, 2) assigned to RGB, respectively, showing lithological variation and structural lineaments in the study area. (**b**) Principal Component Analysis (PCA) image using (PC2, PC3 and PC1) assigned to RGB, enhancing spectral contrast and delineating potential structural features.

FCC provides a visual representation of surface materials by assigning specific bands to RGB channels based on their spectral response (Fig. 3a), which helps in delineating geological contacts by enhancing spectral contrast between different surface materials^21^. In this study, FCC was utilized to visually distinguish between the main lithological units and detect potential fault-related lineaments.

PCA was applied to reduce spectral dimensionality and emphasize data variance, allowing clearer visualization of structural features such as possible faults and lithological boundaries^22^. The first three principal components (PC2, PC3, and PC1), which accounted for most of the spectral variance, (Fig. 3b) were selected for enhancing the interpretation of lineaments.

**Fig. 4:**
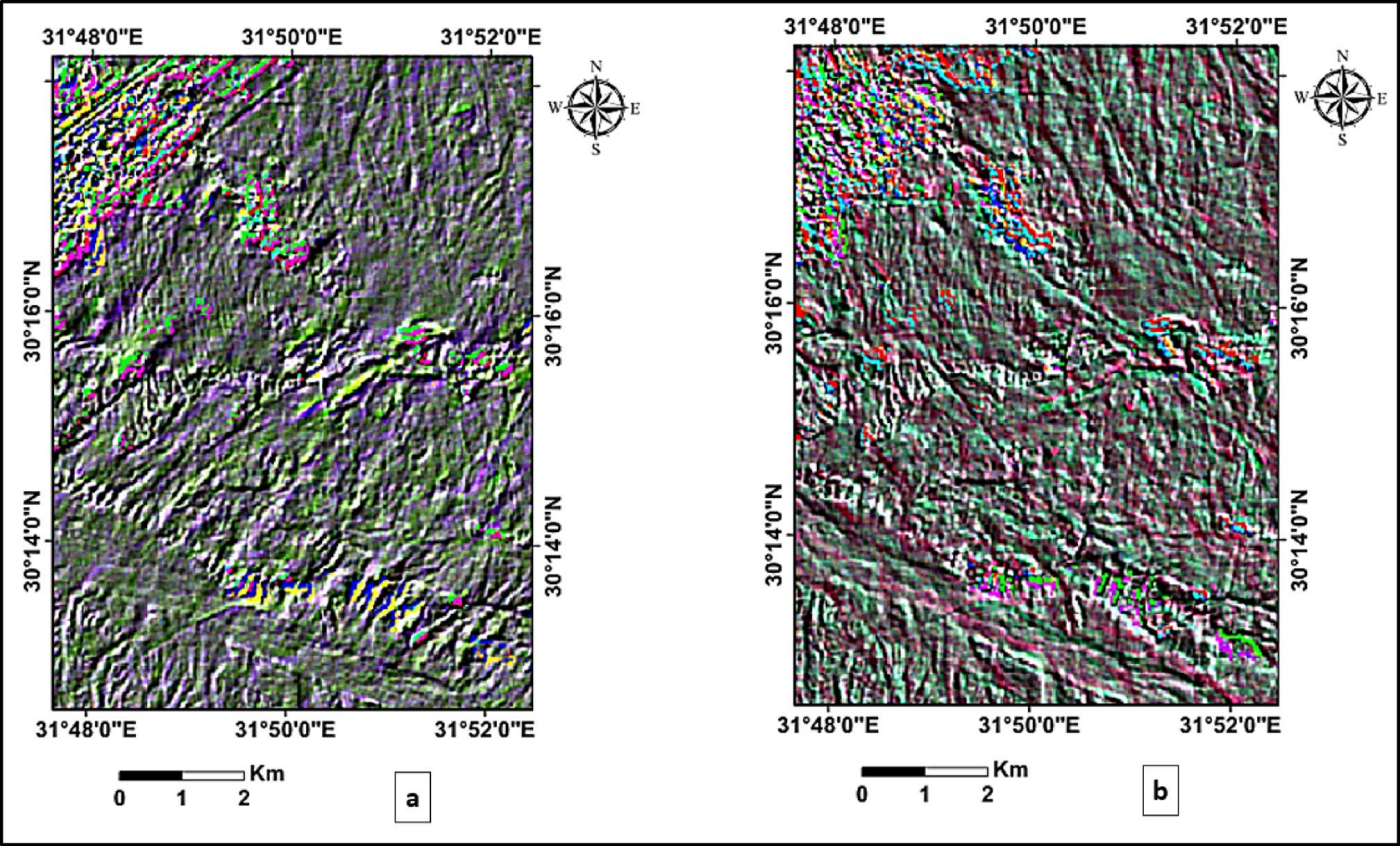
Directional filtering applied to the PCA image at two orientations: (**a**) 45° (NE–SW), and (**b**) 135° (NW–SE), enhancing the visibility of structural lineaments trending in those directions.

To enhance image contrast and improve the visual differentiation of surface features, a (5%) linear contrast stretch was applied to the PCA, FCC, and directionally filtered images. This is one of the most frequently applied transformation, in which the original gray-level or (DN) range of an image is linearly expanded to occupy the full dynamic range of display values^23^. The directional filtering was applied at two orientations (45° and 135°) to emphasize lineaments trending in different directions (Fig. 4).

## Results and validation

### Structural analysis and interpretation

**Fig. 5:**
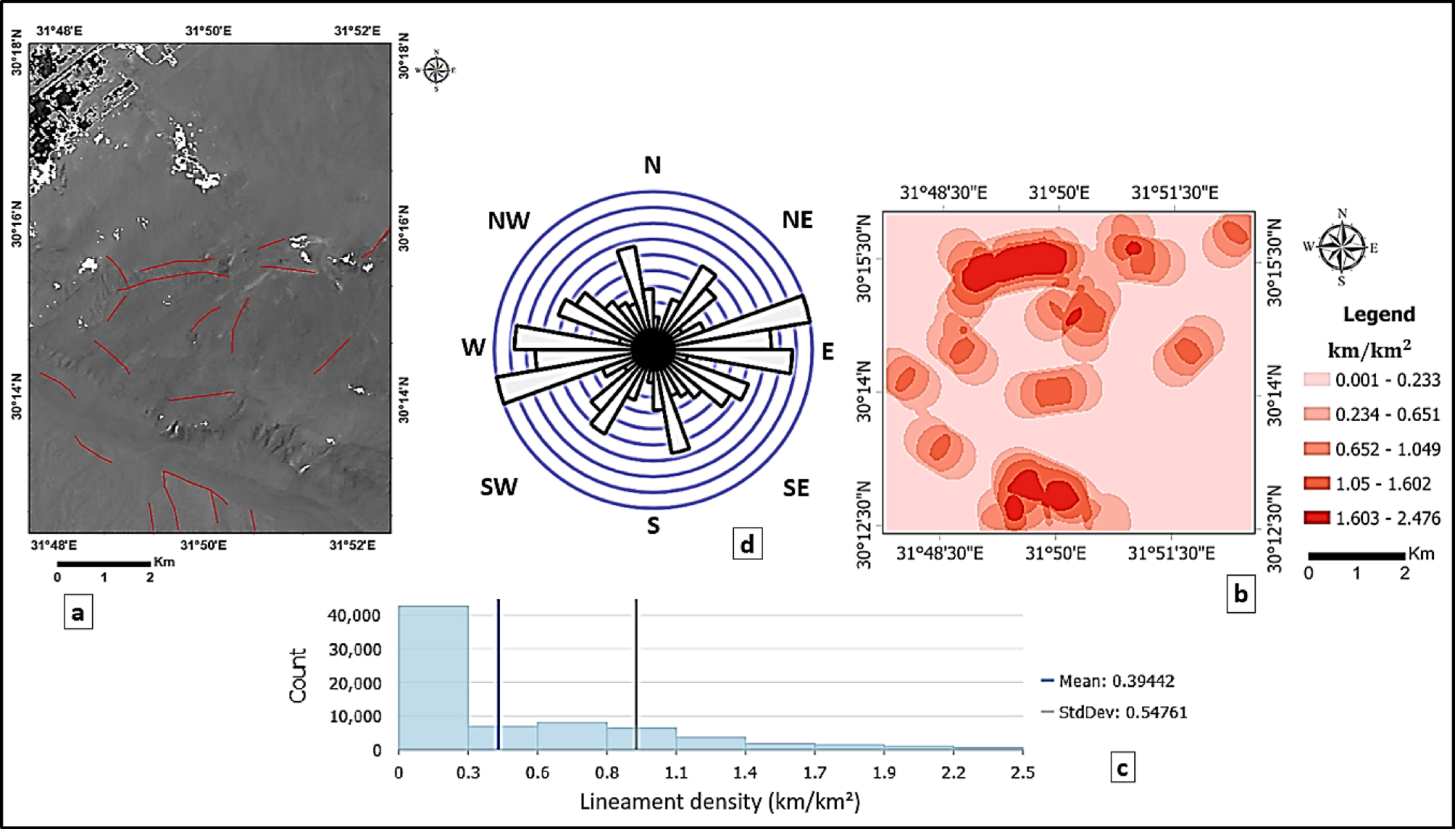
(**a**) PCA-enhanced composite image (PC2 displayed as RGB) with automatically extracted lineaments. (**b**) Corresponding lineament density map (km/km^2^) showing the spatial distribution and concentration of structural features across the study area. (**c**) Histogram of lineament density values displaying their frequency distribution, with mean and standard deviation indicated. (**d**) Rose diagram illustrating the statistically measured orientations of extracted lineaments, revealing dominant NE–SW and NW–SE structural trends.

The structural framework of the study area was extracted and analyzed using principal component analysis (PCA) and directional filtering techniques. Fig. 5a illustrates PCA-enhanced composite image (PC2 displayed as RGB) with automatically extracted lineaments. To ensure the reliability of the extracted lineaments, all results were manually verified through cross-comparison with geological maps, topographic data, and hill-shade imagery in ArcGIS Pro, allowing the removal of non-structural features. Fig. 5b presents the lineament density map (km/km^2^) generated from the validated lineaments and classified using natural breaks, highlighting areas of low (0.001–0.233 km/km^2^), moderate (0.234–1.049 km/km^2^), and high (> 1.05 km/km^2^) structural intensity. The statistical distribution of lineament density values is further illustrated by a histogram with 9 bins (Fig. 5c), providing quantitative support for the spatial variability of structural intensity across the study area. The lineament orientations were quantitatively assessed using a rose diagram with 18 bins (Fig. 5d) based on azimuth measurements, illustrating the full distribution of structural directions across the study area, with NE–SW and NW–SE trends exhibiting the highest frequencies.

**Fig. 6:**
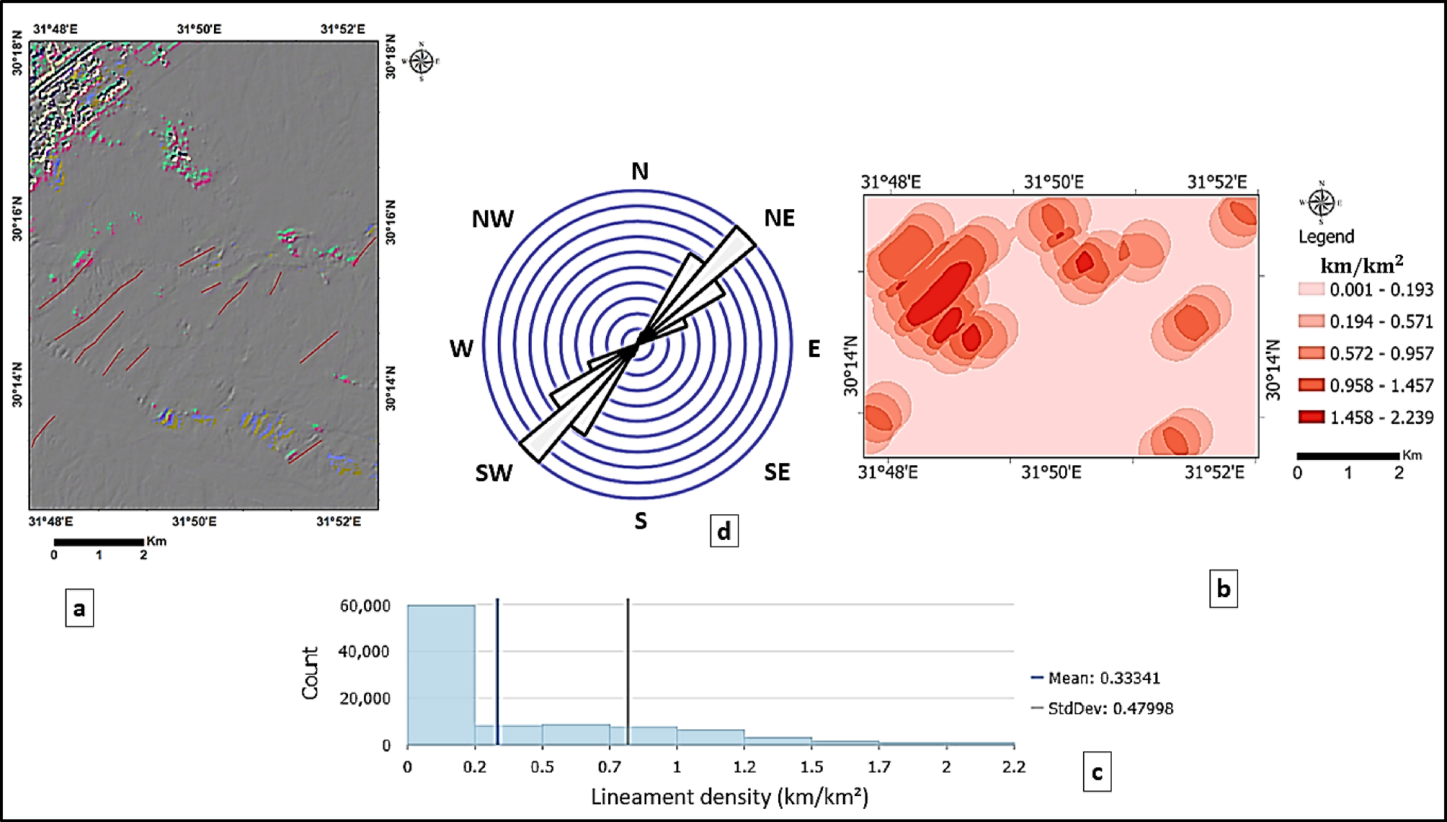
(**a**) Directional filtering at 45°, with overlaid automatic lineaments extracted. (**b**) Corresponding lineament density map (km/km^2^), showing the spatial distribution and concentration of NE–SW–oriented structural features across the study area. (**c**) Histogram of lineament density values, displaying the frequency distribution of NE–SW trends, with mean and standard deviation indicated. (**d**) Rose diagram illustrating the predominance of NE–SW structural trends.

**Fig. 7:**
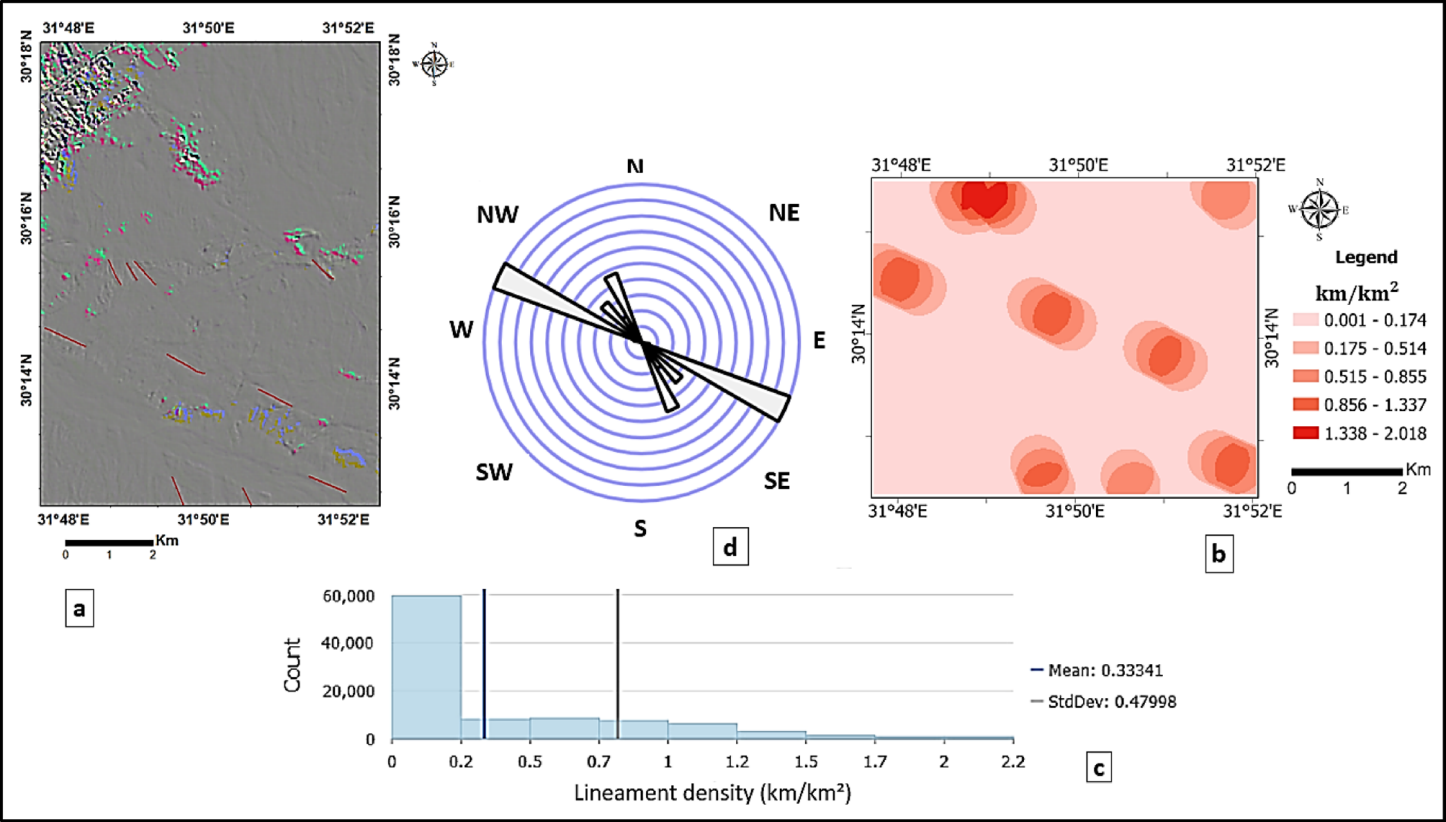
(**a**) Directional filtering at 135°, with overlaid automatic lineaments extracted. (**b**) Corresponding lineament density map (km/km^2^), showing the spatial distribution and concentration of NW–SE–oriented structural features across the study area. (**c**) Histogram of lineament density values, displaying the frequency distribution of NW–SE trends, with mean and standard deviation indicated. (**d**) Rose diagram illustrating the predominance of NW–SE structural trends.

The outputs of directional filters at 45° (Fig. 6a) and 135° (Fig. 7a), overlaid with the corresponding automatically extracted lineaments, reveal the dominant NE–SW and NW–SE structural trends. The lineament density maps (Figs. 6b and 7b) quantify the spatial distribution of these features. For the 45° orientation, lineament density values were classified as low (0.001–0.193 km/km^2^), moderate (0.194–0.957 km/km^2^), and high (> 0.958 km/km^2^), while for the 135° orientation, values were classified as low (0.001–0.174 km/km^2^), moderate (0.175–0.855 km/km^2^), and high (> 0.856 km/km^2^). Frequency histograms (Figs. 6c and 7c) and rose diagrams (Figs. 6d and 7d) provide statistical confirmation of the predominant orientations rather than relying solely on visual inspection. This quantitative approach improves the reliability of fracture detection and facilitates the delineation of structurally controlled zones, particularly along lithological boundaries and wadi systems.

The structural framework identified through this integrated approach provides significant quantitative and tectonic refinements over existing geological data. Specifically, our analysis based on PCA imagery detected a total lineament length of 19.3 km, representing a 25% increase compared to the 15.4 km documented in the Conoco Coral (1987) regional map. This improvement is particularly evident in the detection of minor fractures and secondary structural features. Tectonically, the dominant NE–SW and NW–SE trends are consistent with the regional structural framework of northern Egypt; the NE–SW orientation corresponds to the Syrian Arc system (Late Cretaceous–Early Tertiary compression), while the NW–SE trend is associated with the Red Sea–Gulf of Suez rifting^16^. These structures are crucial for landfill suitability assessment, as their intersection and density define zones of potential geotechnical weakness and enhanced permeability.

### Lithological mapping and validation

#### Hill-shaded DEM with geology

**Fig. 8:**
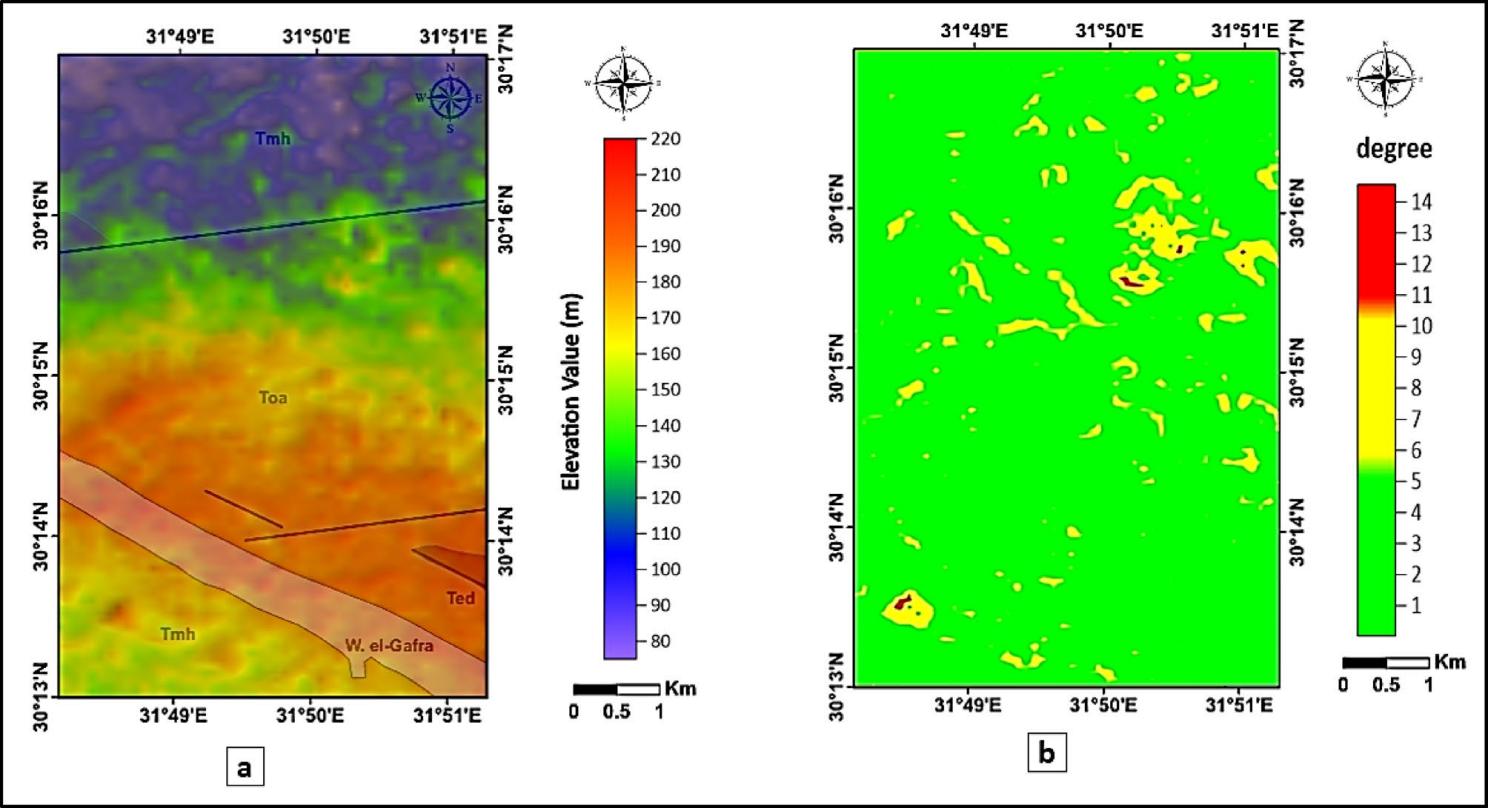
(**a**) Hill-shaded color relief map generated from the DEM, superimposed on the geological map to illustrate lithological boundaries and elevation variations. (**b**) Slope map (in degrees) showing low-gradient surfaces (< 3°) where hill-shade contrast is weak, and higher-gradient zones (8°–14°) that correspond to clearer geological contacts and potential structural-controlled terrain breaks.

To enhance the visualization of lithological boundaries and the influence of topography on surface geology, a hill-shaded color relief map was generated from the ASTER DEM using specific illumination parameters. The hill-shade was produced with a horizontal light angle (azimuth) of 315°, vertical light angle (altitude) of 45°, a Z-scale factor of 2, and an ambient light intensity of 0.022, which collectively optimized the visibility of structural features and terrain breaks. The hill-shaded DEM was then superimposed on the geological map to better illustrate the relationship between lithology and topography across the study area (Fig. 8a). Additionally, a slope map was derived from the DEM (Fig. 8b) to quantify terrain gradients and support the assessment of boundary detectability.

A quantitative and spatial comparison between the mapped lithological boundaries and the enhanced DEM visualization showed a high degree of spatial correspondence. The majority of the geological contacts aligned clearly with slope breaks and topographic transitions evident on the hill-shaded relief. Improvements were particularly observed along the margins of Wadi El-Gafra and in zones affected by Miocene–Oligocene faulting, where the slope values (8°–14°) highlighted structurally controlled terrain variations and enhanced the visibility of lithological breaks. In contrast, minor discrepancies were primarily observed in regions characterized by flat or smoothly eroded surfaces where topographic contrast is inherently limited. Examples include portions of the Hagul Formation (Tmh) and Quaternary deposits, as well as smoother exposures of the Maadi Formation (Ted). In these areas, the low slope values (< 3°) reduce shadow contrast in the hill-shade, making lithological boundaries less distinguishable despite accurate geological mapping. This behavior is expected, as DEM-based shading techniques rely on local elevation differences; therefore, subtle contacts in low relief terrain are less likely to be detected. Overall, the integration of hill-shade and slope DEM derivatives significantly improved the visualization and verification of lithological boundaries, while also clarifying the topographic conditions under which discrepancies are likely to occur.

### Machine learning classification results

**Fig. 9:**
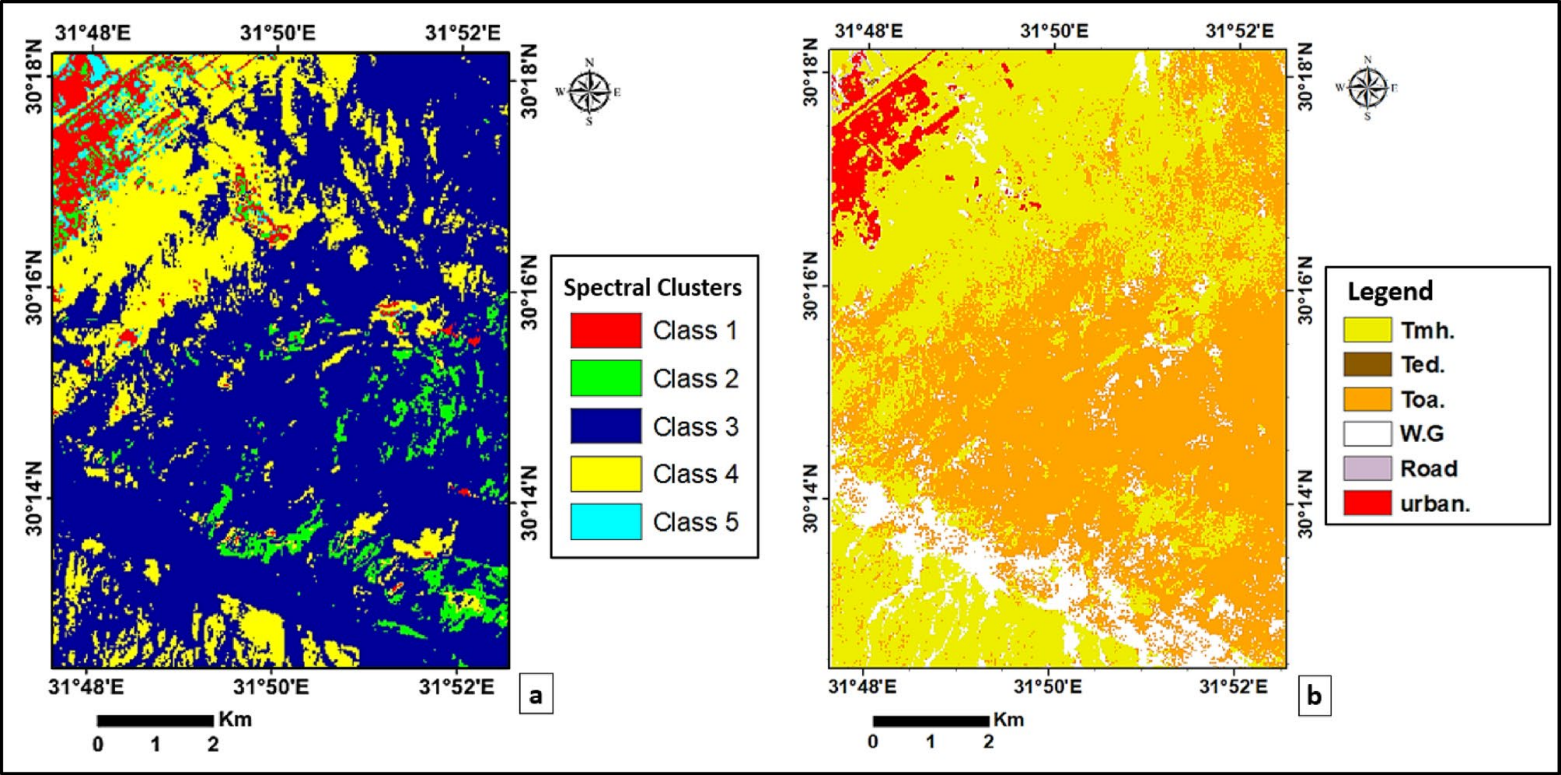
(**a**) K-means unsupervised classification map (five spectral classes) derived from PCA image. (**b**) SVM supervised classification map derived from corrected Landsat 5 TM imagery. Training samples were collected for major lithological units and urban areas within the study area.

The classification results provided complementary insights into lithological discrimination across the study area. K-means groups objects with similar spectral properties into the same cluster while separating those with differing properties^24^, whereas SVM identifies an optimal hyperplane that separates the dataset into distinct classes according to the training samples provided^25^. To determine the optimal number of clusters, several K-means configurations were tested using 4–7 clusters. Each output was visually compared with the published geological map and the PCA-enhanced imagery to evaluate class separability and spectral consistency. The configuration with five clusters produced the most coherent lithological grouping, showing clear spectral boundaries while minimizing excessive mixing between adjacent units. Therefore, the five distinct spectral classes solution (Fig. 9a) was adopted, as it broadly delineated the major lithological variations without prior field-based input and provided a balanced representation of both dominant and transitional lithologies. This approach was effective in highlighting spectral contrasts, although some mixing remained in areas characterized by gradual lithological transitions. In contrast, the SVM supervised classification (Fig. 9b) was applied to the radiometrically and atmospherically corrected Landsat 5 TM imagery. Training samples were carefully selected based on the regional geological map and spectral enhancement techniques (FCC and PCA). This approach provided enhanced spectral contrast between lithological units, allowing for more precise identification of unit boundaries and ensuring representativeness. A total of 44,101 pixels were used as a training dataset, distributed as follows: Toa (21,651), Tmh (15,012), W.G (5615), Urban (837), Ted (735), and Road (251). Subsequently, an independent validation dataset of 13,092 pixels was employed to evaluate the classification performance. The distribution of correctly and incorrectly classified pixels is detailed in the confusion matrix (Table 3) while (Table 4) summarizes the derived accuracy metrics. The results show an Overall Accuracy of 82.49% and a Kappa Coefficient of 0.7235, indicating substantial agreement between the classified map and the ground truth. High producer’s and user’s accuracies were achieved for the majority of lithological units, such as Tmh (84.31% Producer’s Accuracy) and Toa (95.45% Producer’s Accuracy). While classes like Road and Ted exhibited lower accuracies due to spectral mixing and limited spatial extent, the overall output demonstrated clear boundaries and indicated the potential of the SVM algorithm for effective lithological discrimination.

The SVM output demonstrated clearer boundaries and reduced misclassification compared to K-means, reflecting a strong correlation between the classified map and the regional geology. Overall, both the unsupervised and supervised classifications yielded results that closely matched the lithological framework of the study area, confirming the reliability of these algorithms in geological mapping and their potential for effective lithological discrimination by using remotely sensed data.

**Table 3:** Confusion matrix of the SVM classification (Pixels).

	Ground truth							
	Class	Ted	W.G	Tmh	Toa	Road	Urban	Total
Classified	Ted	0	0	0	0	0	0	0
	W.G	0	1076	64	140	0	0	1280
	Tmh	203	149	4482	98	90	76	5098
	Toa	236	409	770	4991	9	1	6416
	Road	0	0	0	0	1	1	2
	Urban	0	0	0	0	47	249	296
	Total	439	1634	5316	5229	147	327	13,092

**Table 4:** Accuracy assessment metrics of the SVM classification results.

Class	Producer’s accuracy (%)	User’s accuracy (%)
Ted	0.00	0.00
W.G	65.85	84.06
Tmh	84.31	87.92
Toa	95.45	77.79
Road	0.68	50.00
Urban	76.15	84.12
Overall Accuracy 82.49%		
Kappa Coefficient 0.7235		

Furthermore, the integration of these machine-learning techniques provided a significant spatial and quantitative refinement over the legacy data. While the regional geological map (1:500,000) generalizes vast areas into uniform lithological units, the 30 m resolution of the SVM and K-means outputs allowed for the discrimination of subtle lithological variations and identified recent urban expansion that occurred after the original map’s publication. This transition from a static, small-scale overview to a high-resolution, updated dataset ensures a more precise representation of the study area’s surface characteristics.

## Discussion

**Fig. 10:**
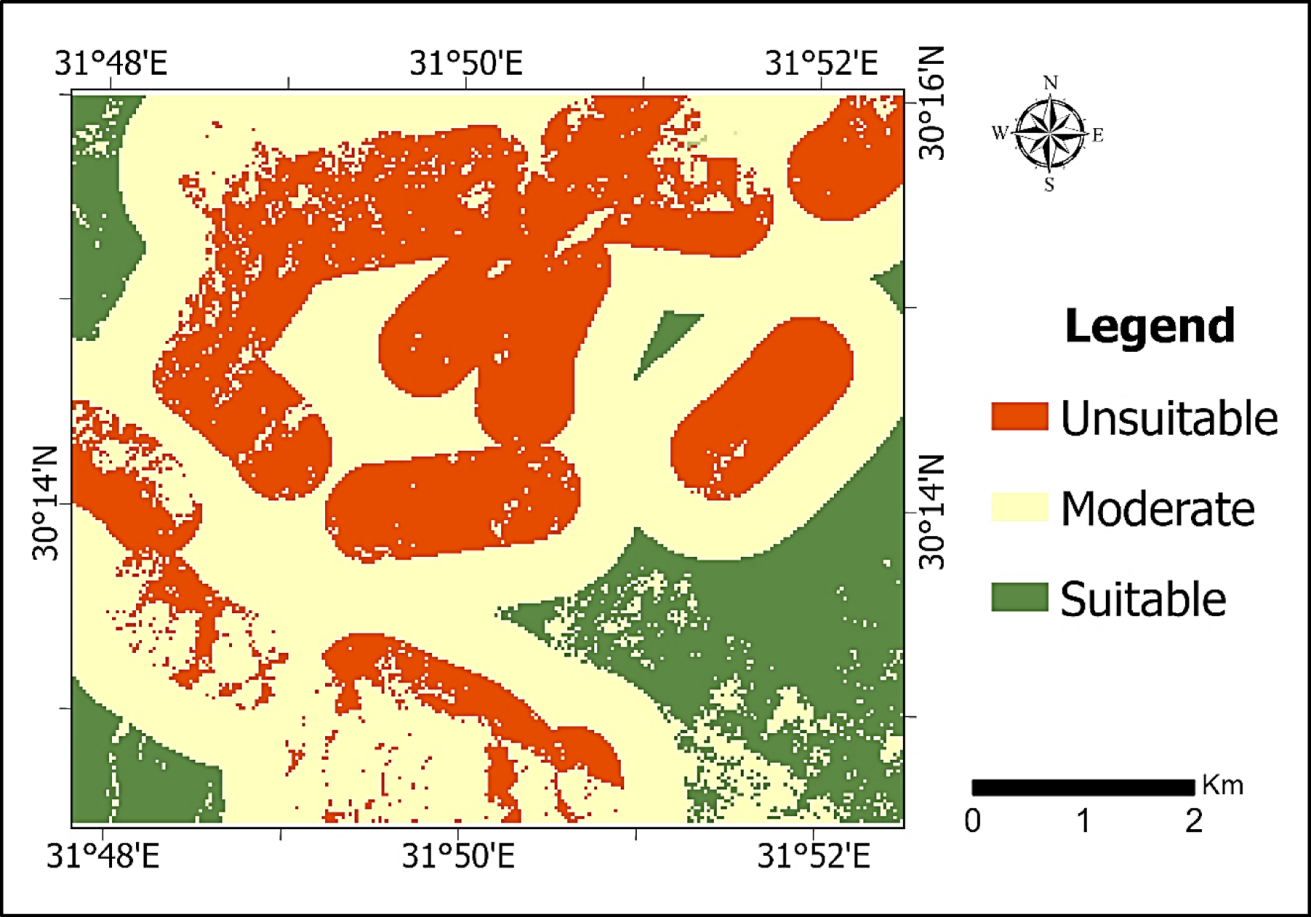
Landfill suitability map.

For a comprehensive evaluation of the geological and anthropogenic factors that control landfill site suitability, the final suitability map (Fig. 10) focuses on the southern sector of the study area, as it represents the most viable candidates for landfill establishment. The northwestern sector was excluded due to high infrastructure density and urban encroachment. Additionally, although the northeastern part is structurally stable, it is dominated by the Tmh Formation (fluvial sands and gravels), which possesses high primary permeability, rendering it less favorable or potentially unsuitable for waste containment. This assessment of surface permeability is further supported by the geotechnical findings reported in the Tenth of Ramadan area^26^ which characterize the Pleistocene sequence as predominantly graded sandy soil and gravels with clays. The presence of such coarse-grained materials justifies the (Low Suitability) score assigned to the Tmh Formation in our model due to its high infiltration potential.

To address the relative importance of the environmental and geological factors, a formal Multi-Criteria Decision Analysis (MCDA) framework was implemented using the Weighted Overlay technique. The criteria selected for the suitability model, along with their assigned weights and scores, are detailed in (Table 5).

**Table 5:** Criteria and weightage.

Criterion	Sub-class	Suitability Score (1–9)	Weight (%)
Lithology (SVM)	Ted Formation (Shale/Marl)	9 (high)	60%
	Tmh & Toa (Sands/Gravels)	3 (low)	
	W. el-Gafra (Wadi deposits)	1 (low)	
Lineament distance (meters)	> 1700	9 (high)	40%
	900–1700	5 (Moderate)	
	< 900	1 (low)	
Constraints	Urban & Infrastructure	Restricted	Excluded

The analysis prioritized geological containment and structural stability to ensure long-term environmental safety. In this context, our prioritization of the Ted Formation (Shale/Marl) as the most suitable lithology (Score 9) aligns with local subsurface investigations that identified shale and clay layers as critical impermeable barriers. According to geophysical logs from the central part of the city, these clay lenses—though discontinuous—act as a protective aquiclude that can impede the vertical migration of contaminants^26^. In addition to lithological Criterion, the structural stability of the study area was a decisive factor in the suitability mapping. The lineaments identified, representing long-term structural features affecting the Eocene to Miocene sedimentary sequences. were analyzed not only as tectonic indicators but as potential flow paths for leachate. Although there is no evidence of recent Holocene activity, they represent potential zones of geotechnical weakness. To ensure long-term environmental safety and the protection of underlying groundwater resources, a conservative buffering approach was implemented. The distance to lineaments was categorized into three suitability levels based on the Natural Breaks (Jenks) method. Areas within 900 m of a lineament (representing the merged first two distribution classes) were assigned low suitability to mitigate the risk of leachate migration through secondary permeability (fracture-controlled) pathways. Moderate suitability was assigned to the 900–1700 m range, while distances greater than 1700 m were classified as highly suitable (Score 9). This precautionary selection of safety margins aligns with established international criteria for landfill siting in similar urbanizing and semi-arid environments^27^, ensuring a robust balance between infrastructure development and hydrogeological protection.

The integrated interpretation of lithology and lineament density demonstrates a direct control on landfill site suitability and environmental safety. Areas characterized by low lineament density and shale/marl-dominated lithology (Ted Formation) represent structurally stable blocks with limited secondary permeability, reducing the risk of preferential leachate migration. In contrast, zones with high lineament density or coarse-grained lithologies (e.g., Tmh Formation) exhibit enhanced infiltration potential and structural weakness, making them unsuitable for long-term waste containment. This explicit coupling between structural integrity and lithological containment provides a geotechnically sound basis for minimizing groundwater contamination risks.

The Multi-Criteria Decision Analysis (MCDA) indicated that approximately 16.2% of the study area as highly suitable for landfill development. To ensure the robustness of these results, a sensitivity analysis was performed by varying the influence of lithology and lineament distance. The analysis showed that even with a ± 10% shift in weights, the primary suitable sites remained spatially consistent, confirming the reliability of the MCDA model.

Furthermore, local hydrogeological data from a nearby well^12^ indicates a groundwater depth of approximately 80 m. This deep-water table, which is consistent with the Pleistocene aquifer levels (60–90 m) reported in regional geophysical studies^26^, significantly reduces the immediate risk of aquifer contamination from potential leachate infiltration, providing an additional layer of environmental safety for the proposed site.

Given the 30 m spatial resolution of Landsat TM imagery, the minimum detectable lineament length is constrained to features exceeding approximately two pixels, as detection reliability in structural mapping is intrinsically linked to pixel f1size^23^. While this resolution is sufficient for regional structural and lithological assessment, smaller-scale fractures and sharp lithological boundaries may not be fully resolved, introducing inherent scale-dependent uncertainty. However, for the scope of regional landfill suitability screening, this resolution remains the most effective standard for identifying major stable blocks and supporting geological safety. This scale of analysis serves as a preliminary regional screening that necessitates future site-specific validation.

Finally, although the SVM classification was based on historical 2002 data, the current analysis (2025) strictly accounts for recent urban expansion through updated (Restricted Zones). By applying a protective buffer around 2025 urban boundaries, the model ensures that the suggested sites remain isolated from future residential growth, mitigating land-use conflicts over the landfill’s operational lifespan.

From a planning perspective, the generated suitability map is intended to serve as a strategic decision-support tool. The ‘Highly Suitable’ zones provide a prioritized zones for detailed site-specific investigations (e.g., geotechnical drilling and hydrogeological testing), while ‘Unsuitable’ zones—due to high lineament density, permeable lithology, or urban proximity—serve as exclusion areas during early-stage planning. This tiered approach enables planners to minimize investigation costs and prioritize geologically secure blocks. By integrating risk assessment into the screening phase, this framework allows for proactive land-use planning, supporting environmental compliance before final site selection and infrastructure investment.

## Conclusion

This study demonstrates the potential effectiveness of integrating remote sensing, machine learning, and GIS-based multi-criteria decision analysis (MCDA) for lithological and structural mapping and landfill site suitability assessment in semi-arid, rapidly urbanizing regions.

The supervised SVM classification applied to Landsat 5 TM imagery achieved an overall accuracy of 82.49% with a Kappa coefficient of 0.7235, confirming the reliability of the lithological discrimination. Automated lineament extraction and density analysis revealed dominant NE–SW and NW–SE structural trends. Notably, the remote sensing-based extraction identified 25% more structural lineaments than those documented in existing geological maps, providing a more refined structural framework for the study area.

The MCDA-based suitability analysis identified approximately 16.2% of the study area as highly suitable for landfill siting. The most favorable zones are characterized by low lineament density, dominance of low-permeability lithologies (Ted Formation), sufficient distance from urban areas and infrastructure, and structurally less fractured conditions. Sensitivity analysis further confirmed the robustness of the model, as the spatial distribution of suitable sites remained consistent under ± 10% weight variations.

From a practical perspective, the proposed framework offers a cost-effective and transferable decision-support tool for urban planners and environmental authorities. By integrating machine learning-derived lithology with structural density analysis, the model supports more informed landfill site selection and contributes to reducing potential environmental risks, particularly groundwater contamination in desert-fringe cities.

Future work could extend this framework by incorporating higher-resolution hyperspectral data to further refine mineralogical variations within the identified clay liners. Furthermore, the high-suitability zones identified in this study provide a prioritized roadmap for subsequent site-specific geophysical investigations and deep borehole logging. These localized engineering studies will be essential for the final design and construction phases, building upon a consistent spatial and structural foundation established in this regional assessment.

## Data Availability

The datasets used in this study are publicly available. Landsat 5 TM imagery was obtained from the United States Geological Survey (USGS) Earth Explorer platform (https://earthexplorer.usgs.gov), and ASTER GDEM v3 elevation data were accessed through Global Mapper software as an online data source originally provided by NASA’s Earth data portal (https://earthdata.nasa.gov). All processed maps and derived results are available from the corresponding author upon reasonable request.
